# The cytohesin guanosine exchange factors (GEFs) are required to promote HGF-mediated renal recovery after acute kidney injury (AKI) in mice

**DOI:** 10.14814/phy2.12442

**Published:** 2015-06-28

**Authors:** Marta M Reviriego-Mendoza, Lorraine C Santy

**Affiliations:** Department of Biochemistry and Molecular Biology, The Pennsylvania State UniversityUniversity Park, Pennsylvania

**Keywords:** AKI, Arf6, cytohesins, epithelial cell migration, Rac1

## Abstract

The lack of current treatment and preventable measures for acute kidney injury (AKI) in hospitalized patients results in an increased mortality rate of up to 80% and elevated health costs. Additionally, if not properly repaired, those who survive AKI may develop fibrosis and long-term kidney damage. The molecular aspects of kidney injury and repair are still uncertain. Hepatocyte growth factor (HGF) promotes recovery of the injured kidney by inducing survival and migration of tubular epithelial cells to repopulate bare tubule areas. HGF-stimulated kidney epithelial cell migration requires the activation of ADP-ribosylation factor 6 (Arf6) and Rac1 via the cytohesin family of Arf-guanine-nucleotide exchange factors (GEFs), in vitro. We used an ischemia and reperfusion injury (IRI) mouse model to analyze the effects of modulating this signaling pathway on kidney recovery. We treated IRI mice with either HGF, the cytohesin inhibitor SecinH3, or a combination of both. As previously reported, HGF treatment promoted rapid improvement of kidney function as evidenced by creatinine (Cre) and blood urea nitrogen (BUN) levels. In contrast, simultaneous treatment with SecinH3 and HGF blocks the ability of HGF to promote kidney recovery. Immunohistochemistry showed that HGF treatment promoted recovery of tubule structure, and had enhanced levels of active, GTP-bound Arf6 and GTP-Rac1. SecinH3 treatment, however, caused a dramatic decrease in GTP-Arf6 and GTP-Rac1 levels when compared to kidney sections from HGF-treated IRI mice. Additionally, SecinH3 counteracted the renal reparative effects of HGF. Our results support the conclusion that cytohesin function is required for HGF-stimulated renal IRI repair.

## Introduction

AKI is defined as a rapid reduction in kidney function and affects 5–9% of hospitalized patients with a greater incidence (>10%) in ICU patients (Chertow et al. 2005; Shema et al. 2009). Several factors contribute to AKI, including intrinsic damage and blockage of the kidney, medications and/or low arterial blood volume (Rahman et al. 2012; House 2013). Such kidney insults cause renal ischemia and reperfusion injury (IRI), an AKI trigger. IRI develops upon reestablishment of blood flow into the kidney after a period of oxygen deprivation. Ischemia and nutrient depletion from the blood promotes an environment where restoration of blood flow (reperfusion) leads to inflammation and toxic waste production, rather than reestablishment of normal function. Consequently, damage and death of tubular epithelial cells occur. These events result in bare tubular areas where only the basement membrane remains (Bonventre and Yang 2011). In order for recovery to occur, the surviving tubule epithelial cells must migrate to repopulate the affected areas of the basement membrane. Subsequently, cell division and differentiation would reconstitute polarity of the epithelial cells (Liu 2002; Bonventre and Zuk 2004).

Several growth factors have been shown to promote recovery of the kidney, including EGF, HGF, IGF-1, and PDGF (Bonventre and Yang 2011). In the kidney, HGF is responsible for maintaining normal function and structure, and is capable of hastening tubule repair and recovery in AKI mouse models (Liu et al. 1999; Fornoni et al. 2001). Studies have shown that this regenerative factor and its receptor c-Met are highly upregulated in kidney epithelial cells upon acute kidney injury to aid in recovery from IRI (Joannidis et al. 1994; Liu et al. 1999; Rabkin et al. 2001). In fact, studies on mice with a deletion of c-Met in kidney tubules suggest that c-Met signaling is essential to protect the kidney after AKI (Zhou et al. 2013). After IRI of the kidney, HGF is believed to prevent apoptosis of injured tubule epithelial kidney cells and induce migration of individual epithelial cells and sheets (Miller et al. 1994; Yang et al. 2003; Dai and Liu 2004; O'Brien et al. 2004; Zhou et al. 2013).

HGF regulates a number of biological processes including cell proliferation and differentiation, motility, organ development, and tissue repair. The c-Met receptor tyrosine kinase, is the HGF receptor. Upon binding, HGF promotes c-Met autophosphorylation and therefore activation of the receptor (Organ and Tsao 2011). The subsequent signaling events following c-Met receptor activation are well established. Activated transduction pathways include Ras, PI3K, Wnt, the STAT, and the Notch pathways. During recovery from AKI this signaling promotes cell survival, migration, and redifferentiation (Birchmeier et al. 2003). To become migratory, cells require members of the Ras family of small GTP-binding proteins. Small GTPases are guanine nucleotide-binding proteins homologous to the *α* subunit of G proteins. They bind to GTP and GDP and act as on and off switches. A number of small GTPases have been found to function downstream of the HGF to promote scattering. These include cdc42, Rho, Rac1, and Arf6, all fundamental players in cell shape and migration. All together, these proteins induce the cell to adopt a migratory phenotype (Ridley et al. 1995; Takaishi et al. 1997; Royal et al. 2000; Palacios et al. 2001; Palacios and D'Souza-Schorey 2003). However, it is not well understood how these proteins interconnect, limiting our ability to specifically modulate the HGF cell response upon kidney damage without affecting other signaling events.

Activation of a small GTPases requires the action of guanine-nucleotide exchange factors (GEF), which will replace the bound GDP for a GTP, thus switching the inactive GTPase to its active form (Gillingham and Munro 2007). Our laboratory has shown that overexpression of cytohesin-2/ARNO, an Arf GEF, in madin-darbin canine kidney epithelial cells (MDCK) cells induces a robust migratory phenotype reminiscent of that of cells treated with HGF (Santy and Casanova 2001). Cytohesin-2/ARNO acts to promote epithelial migration by stimulating the activation of Arf6 and the subsequent activation of Rac1 by Dock180 (Santy et al. 2001, 2005; Santy and Casanova 2002). In addition, we have shown that inhibiting cytohesin-2/ARNO function or Dock180 function impairs HGF-stimulated migration and HGF-stimulated Rac activation in MDCK cells (Attar and Santy 2013). We have therefore demonstrated in vitro that the HGF functions through a cytohesin-dependent Arf6-to-Rac1 signaling module to promote epithelial migration (Attar and Santy 2013).

In this study we have examined the involvement of cytohesin-2/ARNO in HGF-induced kidney repair. We show for the first time that HGF-dependent damaged kidney recovery requires the function of a cytohesin-dependent signaling module. We show that inhibiting cytohesins counteracts HGF-stimulated recovery. In addition, we demonstrate that HGF treatment of ischemic mice promotes the activation of Arf6 and Rac1 in the recovering kidneys in a cytohesin-dependent manner, and that cytohesin activity is required to promote epithelial repopulation of kidney tubules and thus kidney recovery.

## Materials and Methods

### Renal IRI

Protocol number (39526) entitled “Cytohesin dependent ARF to Rac signaling in HGF mediated motility” was approved by the Institutional Animal Care and Use Committee of The Pennsylvania State University on (3/28/2012). Renal IRI was performed as described earlier, with minor modifications (Wang et al. 2009). Briefly, C57BL/6J male mice (7 weeks old, The Jackson Laboratory, Bar Harbor, ME) were anesthetized with pentobarbital sodium (70 mg/kg body weight [bw], intraperitoneally [ip]) and were kept on a heated pad at 36.5°C to prevent hypothermia. To diminish discomfort after surgery, mice were injected with an analgesic (Buprenorphine, 0.3 mg/kg bw, ip). A local anesthetic (Bupivacaine, 4 mg/Kg bw) was also administered subcutaneously (sq) on the incision sites right before surgery. Mice were subjected to dorsal incisions to expose the kidneys, and both renal pedicles were clamped for 26 min (min). Reperfusion was then confirmed visually after release of the clamps. Negative control mice were subjected to the same procedure except the renal pedicles were not clamped (sham operated). Mice were sutured and given 0.5 mL of warm saline (sq), and kept in a warm chamber recovery unit until they regained consciousness.

### Drug treatments

Animals were treated with HGF and SecinH3, singly or in combination. Sham operated and IRI vehicle-untreated mice served as controls. HGF was diluted in injection grade water (0.5 mg/kg bw, ip), and the first dose was administered at 24 h post-IRI, and 24 h thereafter, up to 96 h (Mizuno and Nakamura 2005). SecinH3 (2.5 mmol/L SecinH3 diluted in 50% DMSO containing 2.5% dextrose, 100 *μ*L per mouse, ip) was administered 24 h after IRI, and every 24 h for a period of 4 days (Bill et al. 2010).

### Renal function assessments

Blood samples were drawn from the mice at 0 h of IRI and every 24 h thereafter, and collected in microvettes containing lithium-heparin (Microvette capillary Blood EDTA. Kent Scientific Corp., Torrington, CT). Kidney function was analyzed by measuring the plasma levels of blood urea nitrogen (BUN) (Quanti Chrom Urea Assay Kit, BioAssay Systems, Hayward, CA), and creatinine (cat. no.: DZ072D, Diazyme Labs, Poway, CA). Absorbance was measured using a multimode microplate reader (Synergy 4, BioTek Instruments Inc, Winooski, VT).

### Histology

**Figure 1:**
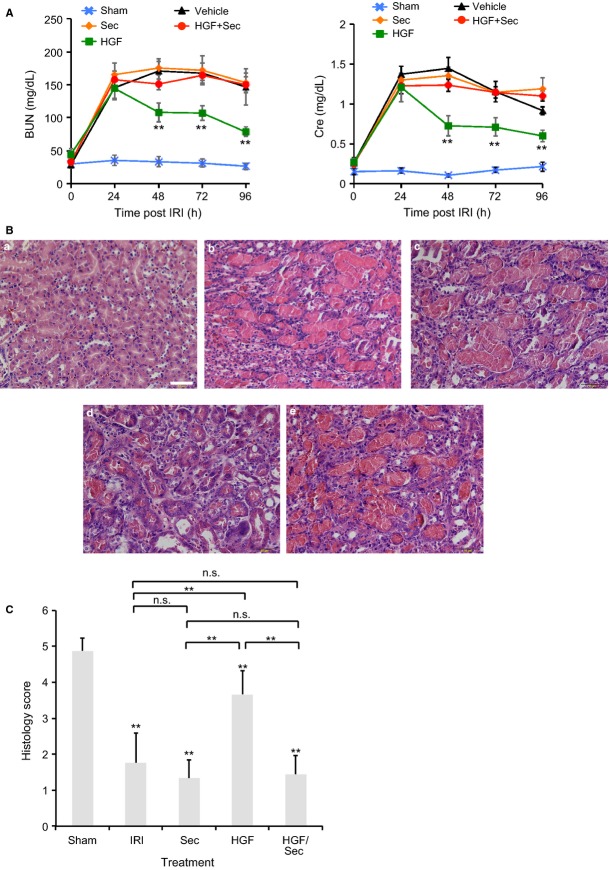
Inhibition of cytohesin activity with SecinH3 impairs HGF-induced kidney recovery. (A) Plasma Cre and BUN levels were measured every 24 h for 96 h in sham-operated and IRI-treated/untreated mice (mice/treatment: 3 sham, 6 vehicle (DMSO), 11 HGF, 9 SecinH3 and 7 HGF+SecinH3). Statistical significance was calculated using an unpaired, two-tailed Student's *t*-test. The vehicle, SecinH3 and HGF+SecinH3-treated mice were not significantly different from each other at any time-point. The HGF treated had significantly improved kidney function when compared to the other injured mice at 48, 72, and 96 h after surgery (**) = *P* < 0.01. The data sets are presented as means ± SE. (B) Representative images of H&E staining of kidney tissue samples from untreated sham-operated mouse (panel a), IRI-untreated mouse (panel b), SecinH3-treated injured mouse (panel c), HGF-treated (panel d), or treated with both HGF and SecinH3 (panel e). Tissue samples were obtained from kidneys harvested 96 h after reperfusion. The scale bars represent 50 *μ*m. (C) Scoring of H&E-stained kidney samples. Sections of H&E-stained kidney tissue were blinded and scored as described in Table1. Data shown are mean ± SD. Sections were from different kidneys: Sham operated (Sham *N* = 8); IRI no treatment (IRI *N* = 17); IRI plus SecinH3 (Sec *N* = 6); IRI plus HGF (HGF *N* = 12); and IRI plus HGF and SecinH3 (HGF/Sec *N* = 9). All IRI samples were significantly different from the sham-operated samples (t-test, indicated by stars on top of the bars). Treatment conditions were compared with a t-test **P < 0.001.

Kidneys were harvested from mice, longitudinally cut in half and immediately fixed in 4% paraformaldehyde for 4 h. Fixed kidneys were embedded in paraffin wax (Leica TP1020 Paraffin Processor and Leica EG1150G paraffin Embedding Unit) and sectioned (5 *μ*m thick) (Shandon Finesse Paraffin Microtome). Standard hematoxylin and eosin staining (H&E) was used to visualize renal structures (Gemini Varistainter). Images were taken using an Olympus BX51. For quantitative analysis, blinded images were assigned a score depending on the level of injury: a value of 1 was given to kidney tissue presenting high degree of damage, and a value of 5 was assigned to a seemingly healthy kidney sections (Table1). Scores were then added up and mean and standard deviation were calculated (Fig.1C).

**Table 1:** A scoring system was used to quantitate the H&E-stained kidney images. Blinded images were assigned a score depending on the degree of damage, where a value of 1 signifies high degree of injured tissue and a score of 5 represents apparently healthy tissue

Score	Parameters
1	Damage. No nuclei around the tubules. Enlarged tubules. Debris in lumen
2	Damage. Few nuclei around the tubules. Enlarged tubules. Debris in lumen
3	Few damaged tubules. Some missing nuclei around the tubules. Some debris in lumen
4	Most nuclei are present around the tubules. No enlarged tubules. Hardly any debris in lumen
5	No apparent damage. Tubules are fully nucleated. No signs of enlargement. No sign of debris in lumen

### Immunohistochemistry

Immunohistochemistry of kidney sections was performed as described earlier, with modifications (The Jackson Laboratory). Briefly, kidneys were fixed with 4% paraformaldehyde for 2 h and transferred to a 18% sucrose solution for 12–16 h. Subsequently, kidneys were placed in a cryomold and embedded in cryomatrix gel (Thermo Scientific, Waltham, MA) prior to freezing. Frozen samples were sectioned (10 *μ*m thick) using a Leica CM1950 Cryostat, and directly mounted on slides for subsequent immunostaining procedures. Kidney sections were allowed to thaw at room temperature (rt), washed once with PBS and fixed with ice-cold acetone for 10 min. Samples were then washed three times with PBS for 5 min each and blocked in PBS containing 1% BSA for 1 h. Next, sections were incubated with primary antibodies specific for Arf6-GTP (Anti-Active Arf6 Mouse Monoclonal Antibody, catalog # 26918 NewEast Biosciences, Malvern, PA) or against Rac1-GTP (Anti-Active Rac1-GTP Mouse Monoclonal Antibody catalog # 26903, NewEast Biosciences) plus or minus primary antibody against aquaporin 1 (Aqp1) (Millipore). After incubation overnight at 4°C with the primary antibodies, tissues were washed three times 5 min each with PBS and incubated with an anti-mouse Alexa Fluor 488 antibody (Jackson ImmunoResearch Laboratories Inc., West Grove, PA) plus or minus anti-rabbit Alexa Fluor 546 (Jackson ImmunoResearch Laboratories Inc) for 1 h at rt. Subsequently, sections were washed three times 5 min each with PBS and, prior mounting tissue was incubated with Alexa Fluor 647 phalloidin (Invitrogen, Grand Island, NY) for 15 min at rt to stain for F-actin.

The efficacy of the anti GTP-Arf6 antibody to specifically detect active Arf6 was tested in vitro by staining of HeLa cells transfected with HA-tagged Arf6-Q67L (GTP Arf6) or with HA-tagged Arf6-T27N (GDP-Arf6) (Fig. S1), and the anti GTP-Rac1 antibody's selectivity has been tested elsewhere (Jiang et al. 2015). Tissues were mounted using Prolong Gold antifade agent (Invitrogen) and visualized in a confocal microscope (Zeiss LSM510, Thornwood, NY). As a control, tissue samples were incubated with the secondary antibody only.

### Statistical analyses

All experiments were performed in triplicate and data sets are presented as means ± SE. Statistical significance was calculated using an unpaired, two-tailed Student's test (Wang et al. 2009).

## Results

### Cytohesin activity is required for HGF-stimulated IRI recovery

HGF promotes recovery of rodent kidneys after IRI in part, by inducing migration of surviving epithelial cells to repopulate the damaged tubular areas (Miller et al. 1994; Ma et al. 2009; Dai et al. 2010; Zhou et al. 2013). Previous studies in our laboratory have demonstrated that cytohesin activity is required for HGF-induced Rac1 activation and wound healing in vitro (Attar and Santy 2013). Furthermore, cytohesin is upregulated 10-fold in kidneys from mice subjected to IRI (Liu et al. 2014). It is then plausible to expect that HGF-mediated damaged kidney repair requires cytohesin function to promote cell migration. To test our predictions we examined whether we could impair the ability of HGF to promote recovery by inhibiting cytohesin with SecinH3, a small molecule inhibitor of cytohesin GEF activity. To do so, we subjected 7-week-old C57BL/6J male mice to IRI and treated them with HGF, SecinH3 or a combination of both 24 h after reperfusion. Blood samples were collected every 24 h for a period of 96 h and levels of BUN and Cre were measured. HGF-treated mice experienced a significant decrease in both BUN and Cre levels 24 h after receiving their first treatment, consistent with previous findings in that HGF promotes kidney recovery (Fig.1A) (Liu et al. 1999; Fornoni et al. 2001). As expected, SecinH3-treated mice showed no improvement in kidney function and levels of Cre and BUN were comparable to those measured in IRI-untreated mice. However, mice receiving simultaneous injections of HGF and SecinH3 did not show an improvement of kidney function, indicating that SecinH3 is antagonizing HGF-mediated recovery (Fig.1A). We next performed histological observations by standard H&E staining of kidney sections from untreated and treated mice that underwent IRI (Fig.1B). When comparing kidney sections from sham-operated mice, samples from animals subjected to IRI showed the expected tubular damage characterized by dilated tubules, loss of epithelial cells, and lumenal debris (Fig.1B, compare panels a and b). As expected, and although still displaying some damage, kidney sections from IRI mice treated with HGF showed a remarkable recuperation of the tubular structures as evidenced by the repopulation of the tubules (Fig.1B, panel d). However, renal tissue sections from IRI mice treated with both HGF and SecinH3 did not present any sign of recovery (Fig.1B, panel e). Kidney tissues from these mice displayed damage to the tubules similar to that observed in untreated IRI mice and mice treated with SecinH3 only (Fig.1B, compare panel e and b, and panel e and c). These results are consistent with our BUN and Cre measurements of kidney function and further support the conclusion that SecinH3 counteracts HGF-stimulated damaged kidney repair, implicating cytohesins in HGF-dependent kidney recovery.

### HGF stimulates Arf6 and Rac1 in injured kidneys and requires cytohesins function

**Figure 2:**
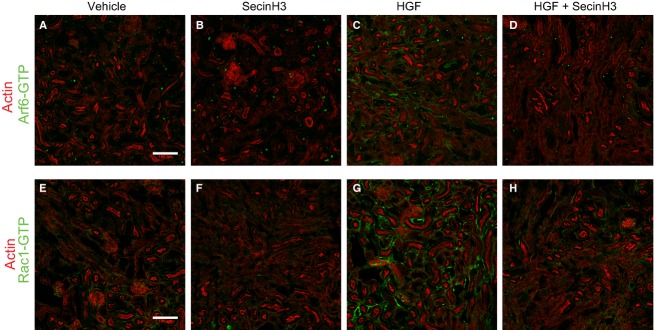
Cytohesins are required to promote HGF-induced activation of Rac1 and Arf6 in injured kidney. Mice were subjected to IRI and treated with either HGF, SecinH3 or a combination of both every 24 h for 96 h. Injured kidneys were then harvested, cryosectioned (10 mm), and stained either against active Arf6 (panels A–D) or GTP-Rac1 (panels E–H), and costained with Alexa Fluor 647 phalloidin to detect F-actin. Tissues derive from: (A and E) IRI-untreated mice, (B and F) SecinH3-treated IRI mice, (C and G) HGF-treated IRI mice or, (D and H) HGF and SecinH3-treated IRI mice. The scale bars represent 100 *μ*m.

**Figure 3:**
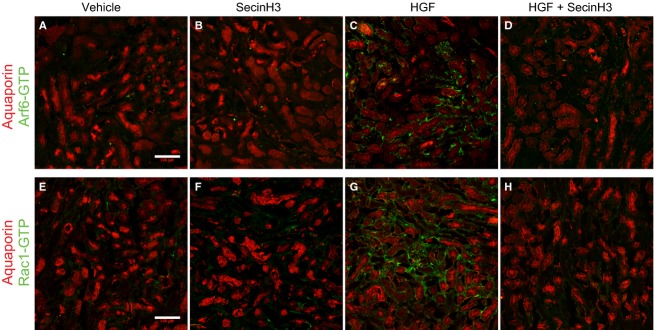
HGF-induced activation of Rac1 and Arf6 in injured kidney occurs mainly at the proximal tubules. Mice were subjected to IRI and treated with either HGF, SecinH3 or a combination of both every 24 h for 96 h. Injured kidneys were then harvested, cryosectioned (10 mm), and incubated with either against active Arf6 (panels A–D) or GTP-Rac1 (panels E–H), and costained with rabbit anti aquaporin-1. Next, sections were stained with anti-mouse Alexa Fluor 488 and anti-rabbit Alexa Fluor 546. Tissues derive from: (A and E) IRI-untreated mice, (B and F) SecinH3-treated IRI mice, (C and G) HGF-treated IRI mice or, (D and H) HGF and SecinH3-treated IRI mice. The scale bars represent 100 *μ*m.

**Figure 4:**
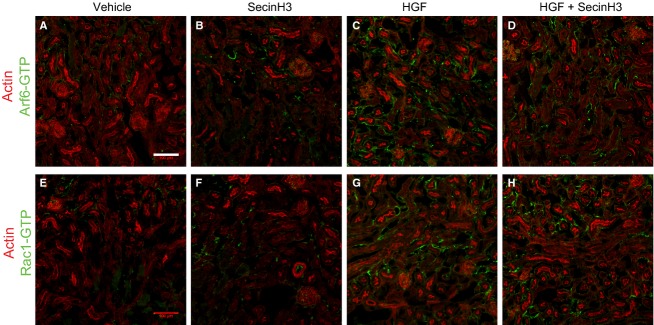
HGF induces the activation of Rac1 and Arf6 in damaged kidneys early after reperfusion. Mice were subjected to IRI and treated with either HGF, SecinH3 or a combination of both 24 h after reperfusion. Injured kidneys were then harvested 24 h after treatment, cryosectioned (10 mm), and stained either against GTP-Arf6 (panels A–D) or GTP-Rac1 (panels E–H), and costained with Alexa Fluor 647 phalloidin to detect F-actin. Tissues derive from: (A and E) IRI-untreated mice, (B and F) SecinH3-treated IRI mice, (C and G) HGF-treated IRI mice or, (D and H) HGF and SecinH3-treated IRI mice. The scale bars represent 100 *μ*m.

Cytohesins act to promote epithelial migration by stimulating the activation of Arf6 and the subsequent activation of Rac1 by Dock180 (Santy et al. 2001, 2005; Santy and Casanova 2002). We have shown that inhibiting cytohesin function or Dock180 function impairs HGF-stimulated migration and HGF-stimulated Rac1 activation in MDCK cells (Attar and Santy 2013). We thus tested whether this signaling cascade is activated in the kidney upon HGF treatment of injured mice. Kidney samples from mice subjected to IRI were collected 96 h after surgery and processed for cryosectioning. Tissues were then immunostained to detect either GTP-bound Rac1 or GTP-bound Arf6, and either actin or the proximal tubule marker aquaporin 1 (Aqp1). Kidney sections from injured mice treated with HGF showed high levels of GTP-Arf6 and GTP-Rac1 in the basal surfaces of proximal tubule cells when compared to untreated IRI mice, demonstrating that HGF does induce activation of both proteins (Fig.2, compare panels c and g with panels a and e). When treating IRI mice with HGF in combination with SecinH3, however, the levels of both GTP-Arf6 and GTP-Rac1 diminish dramatically in the tubules (Fig.2, panels d and h), and are comparable to those of untreated or SecinH3-treated samples (Fig2, compare panels a and e, with panels b and f). Perhaps, SecinH3 treatment alone might reduce the levels of these active proteins even further when compared to levels observed in untreated IRI mice kidney sections (Fig.2, compare panels b and f, with panels a and e). Similar results were observed in tissue samples stained against GTP-Arf6 and GTP-Rac1 and the proximal tubular marker Aqp1 (Fig.3). The beginning of migration and dedifferentiation of surviving tubular cells to reepithelialize the tubules occurs right after inflammation, approximately 48 h after injury (Bonventre 2003). We thus wanted to investigate the levels of active Arf6 and active Rac1 in kidney tissue from injured mice harvested 48 h after surgery. Kidney sections from injured mice treated with HGF experienced high levels of active Arf6 and Rac1 that localized at the basal surfaces of tubular cells (Fig.4, compare panels a and e with panels c and g). To the contrary, kidney samples from injured mice treated with SecinH3 showed reduced levels of these active proteins and were similar to those found in the kidneys from untreated mice (Fig.4, compare panels b and f with panels c and d). Interestingly, kidney tissues from injured mice treated with both HGF and SecinH3 showed a partial reduction in the levels of GTP-bound Arf6 and Rac1 when compared to those of injured kidney tissues from mice treated with HGF only (Fig.4, compare panels d and h with panels c and g). This observation suggests that HGF treatment elicits a rapid response in the pathway that activates Arf6 and subsequently Rac1, and that a single treatment with SecinH3 does not fully inhibit HGF action. Alternatively, it is plausible that early in damaged kidney recovery HGF activates Arf6 and Rac1 through additional cytohesin-independent pathways. Together, these data demonstrate that HGF promotes recovery in part by stimulating the activation of Arf6 by cytohesins. This event leads to activation of Rac1 and migration of renal tubular epithelial cells to repopulate the damaged areas.

## Discussion

Our study reveals for the first time the involvement of cytohesins in AKI repair. The principal repair mechanism of the kidney relies on the regeneration and proliferation of surviving tubular epithelial cells that migrate to repopulate the tubulus. HGF and its receptor c-Met are highly upregulated in the kidney upon acute kidney injury and promote recovery after IRI (Joannidis et al. 1994; Liu et al. 1999; Rabkin et al. 2001). c-Met signaling is in fact essential to protect the kidney after AKI (Zhou et al. 2013). HGF promotes injured kidney recovery in part by promoting tubule epithelial kidney cells migration of individual cells and sheets. HGF-stimulated migration is known to require the activation of several small GTPases including cdc42, RhoA, Rac1, and Arf6 in vitro. Interestingly, however, expressing the activated forms of these GTPases fails to reproduce the robust migration observed in HGF-treated cells (Ridley et al. 1995; Kodama et al. 1999; Palacios et al. 2001). It is possible that the constant activity of these mutants alters the spatiotemporal signaling specificity required for the proper function of the HGF-activated pathway. The cytohesin Arf GEFs promote the activation of Arf6 and subsequently Rac1 to stimulate epithelial migration, and inhibition of cytohesin activity impairs HGF-stimulated migration and Rac activation in vitro (Attar and Santy 2013). Here, we show that by modulating cytohesin function we can alter HGF-mediated response in damaged kidneys. We show that simultaneous treatment of mice subjected to IRI with HGF and the cytohesin inhibitor SecinH3 resulted in diminished HGF-induced repair. Dedifferentiation and migration of epithelial cells approximately occur 48 h postinjury. Thus, as expected, 96 h following IRI, mice that were subjected to IRI and received daily treatment of HGF showed reconstituted tubular structures. However, mice that received both SecinH3 and HGF treatment resulted in no recovery and in a lack of epithelial repopulation of the kidney tubules. We therefore conclude that cytohesin activity is required for HGF-stimulated kidney recovery.

**Figure 5:**
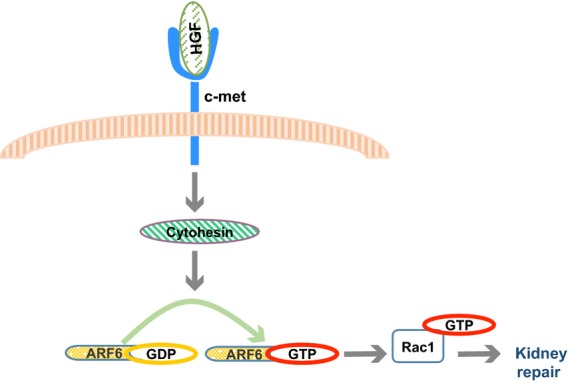
Cytohesin-dependent Arf6 to Rac1 signaling module is required to promote the HGF-stimulated recovery of damaged kidney. Treatment of IRI kidneys with HGF activates the HGF receptor c-met, resulting in a signaling cascade event that leads to activation of the Arf GEFs cytohesins. Active cytohesins promote Arf6 to exchange its bound GDP molecule for GTP, resulting in its activation. GTP-bound Arf6 thus activates Rac1. Activation of Rac1 induces epithelial cell migration to the bare areas of the tubules, promoting damaged kidney recovery.

One mechanism used by cytohesins to induce cell migration is by promoting the activation of Arf6 and the subsequent activation of Rac1. Active Rac1 promotes the accumulation of adhesion molecules and actin at the cell surface (Takaishi et al. 1997). We have shown that the scaffolding protein GRASP assembles a multiprotein complex containing both cytohesins and the Rac1 activating protein, Dock180. Activation of Rac1 downstream of cytohesins requires both the formation of this complex and cytohesin-dependent activation of Arf6. Furthermore, the formation of this complex is required for HGF-stimulated Rac1 activation and migration in vitro (White et al. 2010). Our immunohistochemical analyses indicate that treatment of IRI mice with HGF increases the levels of active, GTP-bound Arf6 and GTP-bound Rac1 in injured kidney tissue, 48 and 96 h postinjury. As expected, the location of these active molecules concentrates at the basal lamina of the epithelial cells, phenotype commonly found in migratory cells. When treating mice with both HGF and SecinH3, however, we observe a decrease in levels of both GTP-bound proteins and impaired recovery of kidney function and structure. Here, we show that HGF promotes kidney injury recovery by activating a cytohesin-dependent Arf6 to Rac1 signaling module. HGF treatment results in the activation of cytohesin, which in turn promotes the activation of Arf6 and, subsequently, activation of Rac1, steps crucial for epithelial cell migration to occur, and thus kidney recovery (Fig.5).

Cytohesins and Arf6 can also promote migration by stimulating the recycling of integrins to the cell surface. Upon activation of the integrin-recycling pathway, cytohesin activity promotes the formation of integrin-loaded trafficking carriers by activating Arf6. Active Arf6 assembles a clathrin and ACAP1 containing coat on recycling endosomes and promotes the recycling of integrins in a Rab11 and actin-dependent manner (Li et al. 2007). We have shown that only certain cytohesin splice variants stimulate integrin recycling. Therefore, it will be of great value to uncover the cytohesin isoform that is necessary to promote HGF-induced migration in damaged kidneys. In addition, it is possible that HGF-stimulated cytohesin activity may coordinate with assembly of new adhesions at the leading edge of migrating cells upon the activation of Rac1.

Migration and dedifferentiation of surviving epithelial cells is the main mechanism of repair after kidney injury, and precedes tubular epithelial cell proliferation and regeneration. HGF enhances kidney repair by in part promoting migration of tubular epithelial cells to repopulate the tubule bare areas (Miller et al. 1994; Ma et al. 2009; Dai et al. 2010; Zhou et al. 2013). We have shown in this report that HGF-stimulated kidney recovery requires the function of cytohesins. Cytohesins are key players in integrin recycling and migration through the activation of Arf6 and Rac1. The spatiotemporal action of these proteins leads to a migratory phenotype in epithelial cells. HGF-stimulated cytohesin activity may coordinate with the assembly of new adhesions at the leading edge of migrating cells upon the activation of Rac1, resulting in reepithelialization of the kidney tubules. We have identified for the first time a link between HGF-stimulated kidney recovery and cytohesin's crucial rule in migration. The study herein demonstrates that a cytohesin-dependent signaling module that leads to the activation of Rac1 is required to promote the HGF-stimulated recovery of damaged kidney. By understanding to what extent cytohesins are involved in kidney repair, we will be able to selectively control HGF motility promoting signaling pathways without affecting other growth factors or altering normal cell functions. As a result, development of new specific targeted drugs to reverse kidney injury will be plausible, and an improvement of mortality rates due to kidney failure will be possible.

## Supporting Information

**Figure S1.** Detection of Arf6-GTP in HeLa cells.
