# Supplementary figures and images for: The cytohesin guanosine exchange factors (GEFs) are required to promote HGF-mediated renal recovery after acute kidney injury (AKI) in mice
Source: Physiol Rep. 2015 Jun 28;3(6):e12442. doi: 10.14814/phy2.12442 (PMC4522160; doi:10.14814/phy2.12442)

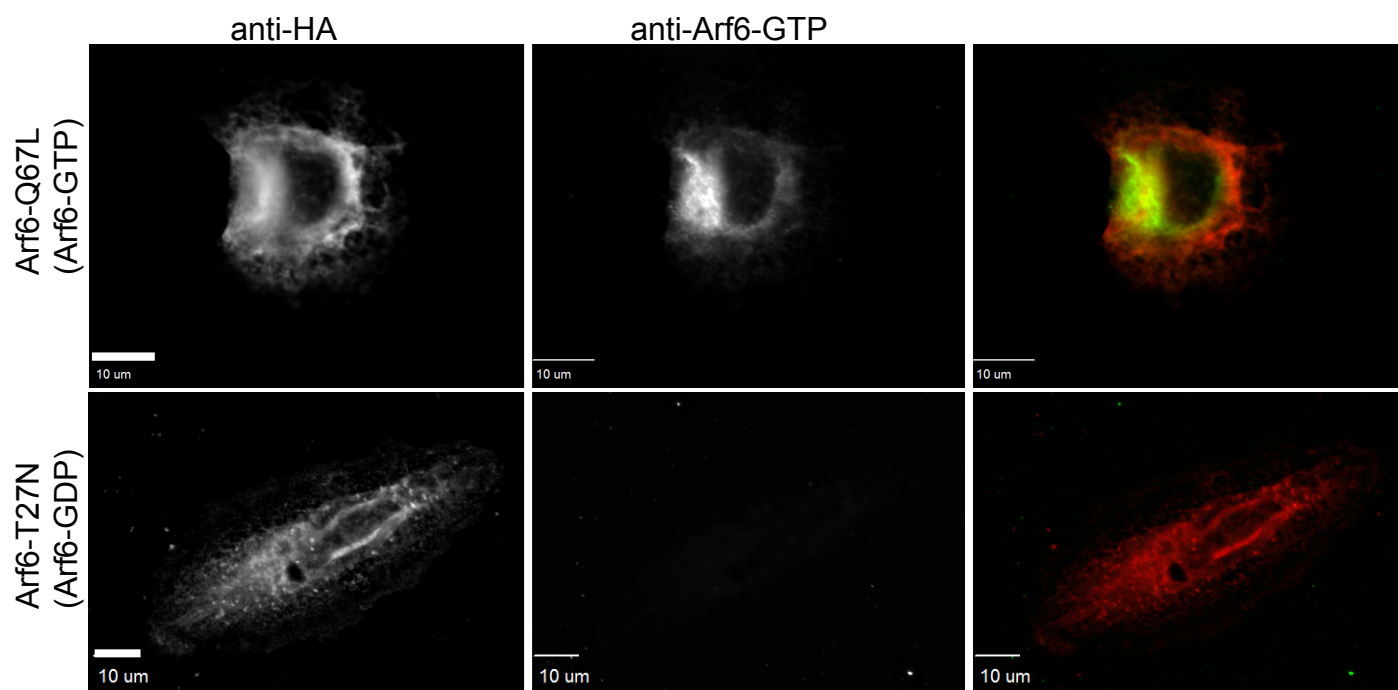

Supplement: Supplementary file 1 — Figure S1. Detection of Arf6-GTP in HeLa cells. [file phy20003-e12442-sd1.pdf]
